# “She is the one who knows”: A qualitative exploration of oral and injectable PrEP as part of a community empowerment approach to HIV prevention among female sex workers in the Dominican Republic and Tanzania

**DOI:** 10.1371/journal.pgph.0000981

**Published:** 2022-09-15

**Authors:** Andrea Mantsios, Ohvia Muraleetharan, Yeycy Donastorg, Martha Perez, Hoisex Gomez, Catherine Shembilu, S. Wilson Beckham, Tahilin Sanchez Karver, Wendy Davis, Samuel Likindikoki, Jessie Mbwambo, Clare Barrington, Deanna Kerrigan

**Affiliations:** 1 Public Health Innovation & Action, New York, New York, United States of America,; 2 Yale School of Public Health, New Haven, Connecticut, United States of America,; 3 Department of Health Policy, Instituto Dominicano de Dermatologia y Cirugia de Piel, Santo Domingo, Dominican Republic,; 4 Department of Psychiatry, Muhimbili University of Health and Allied Sciences, Dar es Salaam, Tanzania,; 5 Department of Health, Behavior and Society, Johns Hopkins University, Baltimore, Maryland, United States of America,; 6 Department of Prevention and Community Health, George Washington University, Washington, DC, United States of America,; 7 Department of Health Behavior, University of North Carolina, Chapel Hill, North Carolina, United States of America

## Abstract

Despite documented interest among female sex workers (FSW), uptake of oral pre-exposure prophylaxis (PrEP) for HIV prevention has been low. Recent trials and regulatory approval of long-acting injectable (LAI) PrEP offer new hope for the potential of this biomedical intervention. We examined FSW’s PrEP-related interest and preferences regarding both oral and LAI PrEP situating these dynamics within their specific social and occupational realities. We conducted this work using qualitative methods across two distinct contexts by conducting 40 in-depth interviews with FSW in Tanzania and the Dominican Republic. Textual data was coded using iterative thematic content analysis. Analytic summaries were developed and reviewed to identify recurring themes. We systematically organized themes within each country and then compared across settings. Women in both settings expressed strong interest in PrEP seeing it as an important option to protect themselves in their work. Most participants preferred LAI PrEP due to expectations of reduced stigma and concerns about daily pill adherence and side effects. Occupational factors such as alcohol use, overnight dates with clients, and fear of violence from clients were identified as barriers to daily oral PrEP. LAI PrEP was seen as having the potential to reduce stress related to oral PrEP. Women who preferred pills discussed fear of needles, skepticism about the injections, and others relayed that taking a daily pill would not be challenging for them. There was a pre-dominant sentiment that women know best whether they are better suited for oral or LAI PrEP. Participants stressed the importance of FSW understanding both options to ensure informed decision-making around PrEP and described community-led peer education as key to delivering trusted information. Community empowerment approaches led by FSW which address structural and psychosocial constraints and promote safe work environments may facilitate equitable access and uptake of PrEP among FSW across settings.

## Background

In lower- and middle-income countries, female sex workers (FSW) continue to be disproportionately impacted by the human immunodeficiency virus (HIV). In both Sub-Saharan Africa (SSA) and Latin America and the Caribbean (LAC), FSW are 12.4 and 12.0 times more likely, respectively, to acquire HIV than women in the general population [1]. Pre-exposure Prophylaxis (PrEP), a daily pill taken to prevent HIV, is a promising biomedical intervention to be added to the HIV prevention toolkit for sex workers. PrEP has been proven to be safe and effective in reducing HIV infection across a range of groups and settings, including key populations such as FSW [2] and has been recommended by the World Health Organization (WHO) since 2015 for all individuals at substantial risk for HIV infection [3]. PrEP access and uptake among FSW has thus far been disappointingly low. The SAPPH-Ire trial in Zimbabwe reported only 38% of FSW (N = 1302) initiated PrEP [4] and a recent study in Uganda documented PrEP uptake among only 55% (n = 158) of eligible FSW [5]. To date, the promise of this highly effective biomedical intervention has not yet been realized.

Barriers to PrEP uptake among FSW occur on several levels. Socio-structural factors, including stigma, discrimination, criminalization, violence, and financial insecurity which have consistently been shown to constrain access to HIV prevention services [6–10], also act as critical barriers to PrEP uptake among FSW. Both the stigma associated with PrEP as a drug for people who have sex with multiple partners and the stigma of being perceived as HIV-positive can deter uptake and adherence among FSW [11–15]. The role of stigma in impeding PrEP uptake among FSW includes fears regarding a lack of privacy in taking or accessing oral PrEP; a fear of disclosure to friends, family, clients, and partners; and worry that discrimination could affect their earnings [11, 16–20]. Widespread misinformation about safety and side effects, distrust in the efficacy of PrEP [21, 22], and the drug’s high cost [11, 15, 23, 24] have also been shown to be key barriers to PrEP among FSW. The need for more frequent HIV testing and clinic appointments is frequently perceived as a barrier to uptake and adherence, in part because this increases the possibility of experiencing sex work- and HIV-related stigma or discrimination enacted by health care providers [14–16, 25–27]. On an individual level, many FSW report that their work schedule makes it difficult to attend more frequent clinic appointments and adhere to taking PrEP [11, 14, 16, 20, 23].

Long-acting injectable (LAI) PrEP has been proposed as one way to mitigate some of these barriers. Phase III randomized trials HPTN 083 and HPTN 084 demonstrated the efficacy and superiority of a long-acting formulation of PrEP, injectable cabotegravir, to prevent HIV acquisition across populations [28, 29]. Specifically, among cisgender women in SSA, HPTN 084 found approximately nine times more incident HIV infections among those receiving daily oral emtricitabine/tenofovir disoproxil fumarate (FTC/TDF tablets) than among those receiving long-acting cabotegravir injections [29] showing that cabotegravir was 89% more effective than daily oral FTC/TDF for PrEP [30]. In December of 2021, the United States Food and Drug Administration (FDA) approved LAI PrEP [31], administered as injections every two months in a clinical setting. European regulatory boards are expected to move forward with approvals as well marking expansion of LAI PrEP to other settings around the globe. Since US approval was received, licensing applications have been filed in Australia, Botswana, Brazil, Kenya, Malawi, South Africa, Uganda, and Zimbabwe [32].

With these advances, there is hope that this new modality can propel HIV prevention efforts among FSW to reduce their dramatically increased risk for HIV across settings. LAI PrEP has the potential to relieve the “burden” of taking a pill every day and address sex work-specific barriers to oral PrEP described above [33]. Multiple studies have found high acceptability and interest in LAI PrEP among FSW, likely due in part to widespread familiarity with injectable contraceptives [11, 34–36].

Previous HIV prevention efforts among FSW have shown that due to the complex socio-structural nature of their HIV risk, programs focused on behavioral or biomedical factors alone, such as health education, condom distribution, or screening for sexually transmitted infections (STI) have limited efficacy [37, 38]. Community empowerment approaches, which seek to transform the social-structural context of sex work and are multi-level in nature, have been found to be more effective and cost-effective for addressing FSW’s specific vulnerabilities to HIV [37, 39–41]. Community empowerment has been recommended by UNAIDS as a best practice in HIV prevention for sex workers for over twenty years [42], and almost a decade ago, the United Nations partnered with the Global Network of Sex Work Projects (NSWP), a sex worker-led umbrella organization, to publish concrete guidance on how to implement interventions using this approach [43]. The approach is centered in the development of social cohesion and the mobilization of the FSW community to promote their health and rights by addressing social exclusion, stigma, and other forms of violence with the use of self-identified solutions and strategic partnerships [37]. Core principles of the community empowerment model are the belief that sex workers themselves are best equipped to identify their priorities and lead responses to these areas of concern and that structural barriers to HIV prevention must be addressed alongside biomedical and behavioral factors in order to sustainably reduce vulnerability to HIV [37, 39].

While there is extensive descriptive literature on the barriers and facilitators to PrEP access, uptake, and adherence among FSW [13, 21, 22], there is limited intervention research on best practices for addressing these factors among FSW. The existing literature on barriers to PrEP access, uptake, and adherence among FSW has led to calls for community-led strategies such as ensuring PrEP is introduced in trusted services tailored for sex workers, that peers play a key role in adherence support, and for a focus on long-term educational efforts to decrease the stigma associated with sex work and PrEP use [14, 44]. With its emphasis on fostering social cohesion, collective action, and strategic partnerships with service providers and other community leaders, the community empowerment model is one of the few that effectively addresses stigma and other structural barriers to PrEP uptake and adherence in a comprehensive manner. One of the best and only examples of a PrEP program employing a community empowerment model is the community-led Ashodaya demonstration project in India [45]. This project included many of the key elements of community empowerment that are effective in addressing the social-structural barriers to oral PrEP described above and was largely successful in promoting high rates of uptake and adherence among FSW, eliminating HIV sero-conversions over a 16-month period [45].

In both Tanzania and the Dominican Republic, the two countries where this study was conducted, oral PrEP is approved but LAI PrEP is not. At the time of this study, however there was no broad access to PrEP in either country, and to date has generally been limited to access via smaller pilot projects [46, 47] At the time of writing, there is a PrEP demonstration project with FSW in Tanzania [48]. According to AVAC’s prepwatch.org country tracker, as of April 2022, the estimated cumulative number of people initiating PrEP is 73,000–74,000 in Tanzania and 2,000–2,500 in the Dominican Republic [49, 50]. Given the substantial HIV risk FSW face and the potential for prevention that PrEP can offer, we would expect to encounter more in the literature from these settings however there is a dearth of research on PrEP among female sex workers in both Tanzania and the DR and none we are aware of around oral versus LAI PrEP. Our study intends to fill this gap by contributing an understanding of the perspectives of FSW in Tanzania and the DR on both oral and LAI PrEP at this critical juncture of PrEP expansion.

As we embark on the next iteration of this biomedical technology and the promise it holds for HIV prevention with this key population, it is critical we explore strategies and approaches for optimal access and uptake. With the advent of LAI PrEP and one that may overcome oral PrEP barriers, there is even more reason now to assess the appropriateness of a community-empowerment approach to ensuring equitable access and uptake of PrEP among FSW communities. In this study, we examined FSW perceptions of and preferences around PrEP and the role of community empowerment approaches in ensuring equitable access and uptake of both oral and LAI PrEP among FSW.

## Methods

### Study sites and participants

This study was embedded in two ongoing cohorts of FSW in each study location which allowed for assessment of interest in and population-specific barriers and facilitators to PrEP among FSW. In the Dominican Republic (DR), FSW (n = 20) were referred to the study by participants and peer navigators from the Abriendo Puertas (Opening Doors) program, an intervention targeting FSW in Santo Domingo, DR. Abriendo Puertas is a community-led multi-level intervention that utilizes an integrated approach to promote HIV prevention and care through individual counselling and education; peer navigation; clinical care provider sensitivity training; and community mobilization [51, 52]. Findings from this intervention revealed that a higher level of engagement with Abriendo Puertas resulted in increased ART adherence (AOR 2.42; 95% CI: 1.23–4.51) and protected sex (AOR: 1.76; 95% CI: 1.09–2.84) [52].

In Tanzania, FSW (n = 20) were part of a larger cohort participating in Project Shikamana (Stick Together), a community empowerment-based combination HIV prevention model for FSW in Iringa, Tanzania. The Shikamana intervention included a community-led drop-in Center as a safe space for FSW to meet as a community and access HIV testing services; venue-based HIV testing and peer educational outreach; provider and police sensitivity trainings; text message reminders to promote engagement and adherence; and peer navigation to support FSW living with HIV in accessing and sustaining care. Results and further details have been previously published [53–55]. At 18-month follow-up, participants in the intervention were significantly less likely to become infected with HIV, with an HIV incidence of 5.0% in the intervention vs. 10.4% in the control arm and decreases in inconsistent condom use over time were significantly greater in the intervention (72.0%−43.6%) vs. control group (68.8%−54.0%) [54].

A total of 40 FSW were purposively sampled [56] from the two settings to participate in the in-depth interviews. We recruited participants through our peer network in each cohort based on sex work venue, type of sex work, and exposure to/engagement in the intervention. We were interested in getting different forms of work as well as different levels of support to understand women’s different needs and then looked for diversity in characteristics such as age as we enrolled participants in the sample. To be eligible for this qualitative study women had to be at least 18 years of age, HIV negative and report having exchanged sex for money in the last month. The two samples varied slightly with regards to number of years in sex work and sex work venue/context of engaging clients. In Tanzania, the majority of women had been engaging in sex work for 5 years or less; three of the women had been in sex work for 10 years or more. In the DR, the sample represented women reporting far more years engaging in sex work with the majority reporting between 10 and 26 years. Tanzania participants were distributed evenly across three types of sex work venues typical to that region (kilabu, grocery, and modern bar). While several Dominican participants reported specific venues where they worked, many were street based and/or contacted by clients via cell phone, Facebook, or WhatsApp (cell phone application).

### Data collection

Semi-structured, in-depth interviews were conducted in confidential spaces at each site by female qualitative research staff who were given additional topical training on PrEP. In both settings, the decision was made to use female qualitative research staff to conduct the interviews based on our experiences with previous studies in which we found this facilitated comfort and rapport building between interviewer and participant. Interviewers were chosen who had prolonged engagement with the study population to increase the rigor and trustworthiness of data collection by having them able to demonstrate an appreciation and understanding of the cultural norms and context of sex workers’ daily lives and thus allow them to potentially obtain more open and honest responses from participants [57].

A semi-structured interview guide with open-ended questions and probes was used to explore specific themes related to HIV prevention and sex work-related stigma but also allowed for flexibility to follow topics that were of interest to participants. PrEP modules were added to the guides and included the interviewer explaining the concepts of oral and LA PrEP and assessing participants’ awareness, perceptions of the advantages, disadvantages, anticipated challenges, and willingness to use. Sample questions from the guide include: What do you think are the advantages/disadvantages of taking oral PrEP for women who exchange sex for money? Are there any concerns or issues that you, personally, have about using oral PrEP? What do you think are the advantages/disadvantages to taking injectable PrEP for women who exchange sex for money? Do you think women who exchange sex for money would prefer the daily pills or the injections every couple of months to prevent HIV? If you were given the choice, would you prefer an injectable or oral form of PrEP? Why? Women were asked about their own perceptions as well as what they perceived to be the opinions of their FSW peers, which at times allowed for richer responses as they answered questions through the lens of peers rather than providing a personal response. Interviews were conducted in the local language (Swahili or Spanish) between 2017–2018 and were approximately 60 minutes in duration.

### Data analysis

All interviews were audio recorded, with participants’ informed consent, transcribed verbatim, and labelled with unique identifiers. An iterative thematic content analysis approach [58] was utilized by two independent coders to analyze the data. In Tanzania, transcripts were translated into English, checked for accuracy by a native speaker, and analyzed by an English speaker. In the DR, transcripts were analyzed directly in Spanish by a Spanish-English bilingual researcher using the English codebook and quotes were translated to English as needed. Both coders engaged in routine notetaking while conducting the analysis which included items to review with the study team in the respective settings such as clarifications in language and nuance within individual interview transcripts. A codebook was developed to inform the coding structure from the *a priori* codes based on the original interview guide and study objectives. Additional domains emerging from the data were evaluated by two independent coders and the study investigators. These were then added to the codebook during the process of refining the thematic coding structure. All textual data was then coded in Atlas.ti [59] to capture both *a priori* and emergent domains of interest [60]. We wrote analytic summaries [61, 62] based on responses to the questions on oral and LA PrEP and reviewed these summaries to identify recurring and important themes. We systematically organized themes within each country and then compared across the settings.

### Ethical considerations

This research was approved by ethical review boards at the Johns Hopkins Bloomberg School of Public Health (USA), the Muhimbili University of Health and Allied Sciences (Tanzania), the National Institute of Medical Research (Tanzania), and the Instituto Dermatologico Y Cirugia De Piel “Dr. Huberto Bogaert Diaz” (IDCP) (DR). Oral informed consent was obtained from all participants prior to their participation. All participants were compensated the local equivalent of $5USD in accordance with community standards of practice in the respective settings for their participation in this study.

## Results

Women in both settings expressed interest in PrEP seeing it as an important option to protect themselves in their work. Most participants preferred LAI PrEP due to expectations of reduced stigma and concerns about daily pill adherence and side effects. Occupational factors such as alcohol use, overnight dates with clients, and fear of violence from clients were identified as barriers to daily PrEP use and LAI PrEP was seen as potentially reducing stress related to oral PrEP. Women who preferred pills discussed fear of needles, skepticism about the injections, and others simply stated taking a daily pill would not be challenging for them. There was a pre-dominant sentiment that women know best whether they are better suited for oral or LAI PrEP. Participants stressed the importance of FSW understanding both options and ensuring empowered decision-making around PrEP. Some differences emerged between the two settings including that Tanzanian women were uniquely resistant to the idea of taking a daily pill if they were not ill and they expressed more concern than their Dominican counterparts around stigma associated with pill taking.

### Overall interest in PrEP and PrEP acceptability

Women in both settings expressed interest in PrEP seeing it as an important option for FSW to be able to protect themselves in their work. Before the interviewer described the long-acting injectable formulation, participants talked at length about the benefits of a pill to prevent HIV given ongoing risk in the context of their work. A salient theme in both settings was the appeal of PrEP providing protection in the common scenario of a client refusing to wear a condom or a condom breaking during sex. One Tanzanian participant described it as follows:

I think it will be a good thing, because you might get a client that you have never met before, you agree that you will use protection but when you get in, he refuses but if you have taken the tablets, you will not be infected.

(Tanzanian sex worker)

A Dominican participant echoed this sentiment saying:

They [fellow FSW] will be happy because they would be taking it [oral PrEP] every day, and for example, if they have a sexual relationship without a condom or the condom breaks, they will not get it [HIV].

(Dominican sex worker)

Both Tanzanian and Dominican participants described the advantage of PrEP safeguarding against HIV infection specifically in the case of a client intentionally breaking a condom, a common occurrence, as described by participants. As a Tanzanian woman described:

One might put on a condom but tear it intentionally so as to purposely infect you. Little does he know that you already took a tablet so you cannot get the infection.

(Tanzanian sex worker)

Amidst the mostly positive reaction, some women in Tanzania raised the question of why they would take pills if they were not sick. As one participant said, “Do you think I want that work of taking pills every day while I do not have AIDS?” (Tanzanian sex worker). Another participant echoed this sentiment saying, “Someone will find herself like ‘dah! So I am taking medicine every day. Have I already become like an infected person or what?’” (Tanzanian sex worker)

### Preference for LA PrEP

After the interviewer described LA PrEP, most women in both settings expressed a clear preference for this option. Their preference for LA PrEP was based on both psychosocial factors (e.g., relief from worrying about missed pills, fear of side effects associated with pills, and reduced opportunities for stigma associated with pills) and structural factors in their work environment (e.g., fear of violence from clients associated with pill use, and difficulty taking pills due to alcohol use and overnight dates with clients).

### Relieves stress of worrying about missed pills

Women in both settings felt that adhering to oral PrEP would be difficult because of the daily burden of remembering to take a pill. Participants identified that LA PrEP would provide relief from the stress of worrying about missed pills and peace of mind associated with a logistically more manageable form of administration for their lives. One Tanzanian participant described it as follows:

The benefit of injection is that you are free, different from using pills. That if you don’t take it, when you forget you start having worries that “now I didn’t take the pill and I have done this, I am already infected” rather than when you get an injection you understand that, at a certain date, after two months I am supposed to go and get injected.

(Tanzanian sex worker)

Alluding to the stress of having to remember pills, participants described a sense of freedom and peace of mind they would be afforded by the injectable option. As a Dominican participant described:

I would be interested in the injection.… Because an injection is already given to you, you already know that for two months you will be calm.

(Dominican sex worker)

A Tanzanian participant echoed the sentiment that adhering to an injection would be logistically feasible and offer peace of mind saying:

The injection is in the body at all times. I just remember that on a certain date, even if I wake up in the morning, I will have that thing of mine which they document the date you need to go back to get a shot [appointment reminder card]; I have it. On the specific date, I go and get a shot. I get the shot and continue with my life.

(Tanzanian sex worker)

### Fear of side effects associated with pills

Both Tanzanian and Dominican participants described fear of side effects associated with pills as being a barrier to oral PrEP use drawing a parallel to side effects women experience when taking oral contraceptives. A Tanzanian participant described it as follows:

Many will prefer the injection. It is similar to the one used for family planning, right? Many of them dislike the tablets, they prefer the injection and the implants because they say the ones taken orally have disturbances [side effects].

(Tanzanian sex worker)

Similarly, a Dominican participant said:

Well, an example is the planning pill that makes your menstruation go down a lot because they make you fat or give you a tachycardia and stuff…if it [oral PrEP] causes any of those things, that is the only bad thing that it would see because the drugs do not prove the same to everyone.

(Dominican sex worker)

A few participants noted the possibility for side effects with an injection as well though they were generally perceived as less severe, as expressed in the following quote:

It is true that with pills, you can get those side effects. And even with injection, you can get injected and feel pain or sometimes it can bring about nausea and pain. I mean small side effects.

(Tanzanian sex worker)

A Dominican participant articulated that given her concerns about side effects from medicines in general, her preference would be to learn about any side effects associated with oral or LA PrEP before making a decision about her preferred modality:

I would be interested to know more or less what reactions the pill gives?.…If it doesn’t give me any reaction, it’s good, because it won’t do any harm, the better it will prevent me from a disease. Because sometimes you take a medicine and you get dizzy, nauseous, headache…That is why I would like to know about the pill and the injection which would be the best product.

(Dominican sex worker)

### Reduced opportunities for stigma

Among Tanzanian participants, LAI PrEP was perceived by most women as a more discrete option that would reduce opportunities for stigma around pill use. Several women in Tanzania described the appeal of LAI PrEP specifically because getting an injection seemed more private to them as they described difficulty concealing pills and fear of stigma associated with pills if they were to be discovered.

Many [FSW] will prefer injection. Nobody likes to see you carrying around those pills in your bag, they will think you are infected but if you get an injection nobody will suspect anything.

(Tanzanian sex worker)

Speaking specifically about potential for stigma from sex worker peers, one participant said:

With pills, I am supposed to take one every day…they will see injection is better than pills… someone can start wondering where to keep my tin, the place to store that tin. Because you can be surprised there are other FSW at a bar, they have one room which they are given to sleep in. Now she thinks, let me not put it carelessly where my friends can see it.

(Tanzanian sex worker)

Participants in the DR did not voice the same concerns about potential stigma associated with PrEP pills or the idea that reduced stigma was a specific benefit of LAI PrEP.

### Difficulty taking pills due to alcohol use and overnight clients

Several structural factors were described as influencing women’s preference for LAI PrEP including daily realities of their work. Among Tanzanian participants, a barrier to oral PrEP discussed by several women was the reality that heavy alcohol use in their community would present a challenge for women regularly taking pills. As one woman described:

The injection, because if you take the tablet daily…you know, the people who work at the bar, she might wake up in the morning and drink alcohol all the way until the time to close the bar. When will she remember to go and take her medicine?

(Tanzanian sex worker)

Several women in both Tanzania and the DR noted that LAI PrEP could help address the challenge of remembering pills amidst the realities of their work. A Dominican participant described it as follows:

It’s [the injection] better than daily [pills]. The day I go to bed drunk and I don’t want to pick up that pill, it’s going to go to the devil because I’m not going to take it [laughs]. To me, no pill crosses my mind but the injection I will be pending for the day of that month I have an appointment.

(Dominican sex worker)

Participants also described the challenge of adhering to daily pills given the often spontaneous nature of their work, including unexpectedly traveling and staying overnight with clients. A Tanzanian participant described this as follows:

Sometimes you are with someone but he won’t let you go till the sunrise, even if you ask him to leave and go see the family he would say he has money, “you will go later,” so if you have not carried the medicine then you are at loss.

(Tanzanian sex worker)

### Violence from clients

Participants in both setting were concerned that carrying pills which could be discovered by clients would put them at risk for violence, stigma, and/or refusal to pay for sex. Two Tanzanian participants described anticipated violence from PrEP disclosure as follows:

You have taken a client and you have pills. First of all, clients might even kill you. He might tell you, “you are taking medicine. Do you want to kill me? So you are an infected person!” You see?

(Tanzanian sex worker)

If you meet a client and he sees the tablets he might think you are infected and he might beat you or refuse to give you money without knowing what the tablets are for.

(Tanzanian sex worker)

Dominican participants also described the challenge they would face with clients assuming pills were antiretrovirals (ARVs) and the stigma and potential violence that could result from clients believing they were HIV infected.

No matter how much I tell him no, I’m taking it [the pill] to avoid it [HIV], no, he won’t believe it because he will say, “you have it [HIV], you have your illness and you are taking it [the pill] because you are sick.”

(Dominican sex worker)

### Preference for pills

Despite preference for LAI PrEP expressed by most participants, a handful of women said they would still prefer to take pills orally over receiving an injection. Reasons included fear of needles, skepticism about what would be injected, and others simply stated taking a daily pill would not be challenging for them. A Dominican woman spoke about women who may have a fear of needles as she does:

There are many [FSW] who would take it [LAI PrEP] but there are others who would not, same as me, because they are afraid of the injection. I’m afraid of it myself. I don’t know, but it gives me something; when I go to the hospital, I close my eyes to avoid seeing the puncture.

(Dominican sex worker)

A Tanzanian participant expressed her preference for pills due to doubts and skepticism about the injection:

I would just choose the tablet because with the tablet, you just take it and drink it; that’s it. Now, with the injection, I don’t know, inject after every two months. Now, you might never know. Perhaps they give you saying it is an injection but they infect you. How would you know?”

(Tanzanian sex worker)

Participants in both settings who favored pills discounted the possibility for stigma associated with pill use drawing parallels to strategies women employ to conceal other medicines. A Tanzanian participant compared taking oral PrEP to taking ARVs:

For us women, you know the time you are supposed to take it and not necessarily for anyone to see you. You hide your tin, you take it in confidence, no one will know. There won’t be any difficulties, it will be the same as taking ARV. Others work in bars and their friends don’t know, they take their medication in confidence. There won’t be any problem.

(Tanzanian sex worker)

A Dominican participant drawing a parallel to concealing her use of oral contraceptives said, “Well it depends because if that pill is for health, I don’t have to take it in public. That’s just like I take a pill to not get pregnant, no one has to know what that pill is for.” (Dominican sex worker)

### The importance of options

Women in both settings talked about the importance of FSW having options and being educated about them so that women interested in PrEP can choose which modality they prefer. Despite most participants voicing preference for LAI PrEP, women in both settings felt it was important to acknowledge that personal preferences vary from individual to individual and women know best whether oral or LAI PrEP would be better suited for them. A Tanzanian participant put it simply as:

There are some who will say, “Ah! For me, the injection is better.” Other might say the tablets are better. Just like that. People are diverse.

(Tanzanian sex worker)

Another said, “One prefers pills to injection, another prefers injection to pills. So, that is why it is different. The one using pills and the one using injection are different.” (Tanzanian sex worker)

Reflecting the importance of a menu of options, participants in the DR thinking about which form of PrEP sex work colleagues would prefer said,

She is the one who knows which of the two [pills or injection]. There are people who are afraid of the spike [getting a shot]…there are people that I have seen that when they take the pill, their stomach hurts.

(Dominican sex worker)

Well there are people who do not like to inject themselves, they are terrified of it, and obviously those people are going to prefer their pills, but those who do not mind being [injected], would prefer that so as not to have the commitment to take it all the time.

(Dominican sex worker)

### Community education and empowered decision making

Participants in both settings spoke about utilizing community education strategies to ensure clients, partners, and fellow sex workers are educated about PrEP. A Tanzanian participant described the opportunity to educate a client as follows:

Now you will advise him. You don’t tell him that you are using but you advise him “listen, do you know there is protection against AIDS?” “I don’t know.” “It is there.” “What?” “There are pills which you swallow everyday.” Another one opens up there, another one opens up, so there you help the community as well, in informing them.

(Tanzanian sex worker)

Some participants suggested disclosing their own PrEP use could help others. A Dominican woman said:

Yes, they are going to think what is it that I have. I would tell him what they are for, I would tell him […] so that I don’t get HIV, that I’m taking that medicine, that anything that prevents me from not getting infected.

(Dominican sex worker)

Tanzanian participants focused on the importance of educating FSW about PrEP specifically to counter the potential for stigma and negative associations with ARVs. One woman described it as follows,

Just like the ARVs, as people use them, there is still stigma. They are still being stigmatized. So we do not know how it (PrEP) will be when it also arrives. But I still go back to [the idea] that it is up to someone as an individual and the education that that person gets.

(Tanzanian sex worker)

Another woman spoke about the complexities around ARV use among FSW that would complicate how PrEP is received and the need this underscored for education. She explained this as follows,

I personally will see the benefits but them (other FSW), they are just so stubborn maybe until they get a proper education, they will be stubborn. They will be like “If those ARVs failed, why not these? If that cure failed, will this be effective for prevention?” Some of them might come pick up the pills but not take them […] They might agree to take the pills, because so many of my fellows doing this job agree to take those ARVs then throw them under their beds when they get home. So even these others should get some proper education and they should understand that these drugs are helpful and they should agree.

(Tanzanian sex worker)

Most women were interested in community education around PrEP both for themselves and for their fellow sex workers and clients and described it happening via one-to-one peer education, as described in the quotes above, rather than in a more formal format.

## Discussion

Despite psychosocial and structural level factors presenting barriers to PrEP uptake [11–16], we found strong interest in this biomedical HIV prevention intervention among FSW in both study settings. HIV and sex-work related stigma along with realities of work conditions and environment are among the most pervasive factors creating barriers to oral PrEP among FSW, making LAI PrEP particularly appealing as it shows promise to overcome some of these challenges. Our findings show that while most FSW were interested in LAI PrEP, some would still prefer to take pills. More importantly however, women conveyed the importance of knowing about both options to be able to make an informed decision about the best PrEP modality for them asserting that in the end, women know best what will work for them and need to be given options so they can choose. These findings underscore the importance of creating awareness and understanding of PrEP options to ensure empowered decision-making by FSW around PrEP as an HIV prevention option. Community empowerment approaches led by FSW which address structural and psychosocial constraints and promote safe work environments may facilitate equitable access and uptake of PrEP among FSW across settings.

The success of community empowerment approaches used in behavioral interventions among FSW provides solid evidence for the effectiveness of their use by FSW communities. The comprehensive nature of community empowerment approaches more easily allows for the integration of PrEP than other models; however, it is important to ensure the same guiding principles are followed for the roll-out of this biomedical intervention as have been applied for behavioral interventions in the past. These guiding principles are developed by sex workers themselves and acknowledge that sex worker communities are the best equipped to decide how PrEP might be integrated into their lives and current health protection strategies, what barriers or challenges they might encounter in accessing and adhering to PrEP, and how they as a community should address these barriers [15, 37, 39, 43, 55].

The goals of a community empowerment approach to PrEP roll-out would be for sex worker communities to employ solidarity and capacity building to advocate for their rights to PrEP, increase community knowledge and decision-making related to PrEP, and collectively address barriers to accessing and adhering to PrEP [15]. This process could include the following: disseminating information on PrEP throughout the community, with the use of peer educators and venue-based outreach; forming groups to develop community strategies for accessing and adhering to PrEP; employing peer navigators or forming support groups to assist with PrEP adherence; and establishing relationships with service providers to ensure non-judgmental and effective care related to PrEP [15, 43, 55, 63]. Early and regular relationship building with key governmental officials and service providers is critical to addressing structural barriers to PrEP and ensuring the sustainability and external acceptability [55]. As emphasized by participants in this study, community ownership and leadership must be at the forefront, and all activities must be shaped by FSW priorities [15].

A key tenet of community empowerment approaches is the right to work in a safe working environment free of stigma and violence. The findings from this study reveal daily realities of sex work including experiences of stigma, sexual and physical violence, and heavy alcohol use in sex work venues which women raised in the context of considering how oral and injectable PrEP would play out in their lives. This context of environmental risk cannot be addressed by PrEP but rather should be addressed in parallel with PrEP being an available prevention option. A community empowerment approach is based on the premise that structural barriers to HIV prevention must be addressed alongside biomedical and behavioral factors in order to sustainably reduce vulnerability to HIV [37, 39]. Study findings speak to the need to transform the social-structural context of sex work while ensuring sex workers are aware of and making informed and empowered decisions around if PrEP is a suitable HIV prevention option for them.

Community empowerment approaches are based in the principle that sex workers should have options and make their own decisions about their health and well-being as opposed to interventions which direct behavior. Women in this study stressed the importance of FSW understanding both oral and LA PrEP and the role of community-led peer education in ensuring empowered decision-making around PrEP. Participants called for educating peers, partners, and clients about PrEP as a strategy for reducing stigma. Previous studies have also shown that FSW believe increased awareness of PrEP among the general public is necessary to minimize stigma and facilitate access and adherence to PrEP [12, 20, 25]. Specifically, educating communities about HIV treatment versus prevention has been identified by FSW as critical to PrEP roll out [64, 65].

A key step for integrating PrEP within a community empowerment approach is disseminating information and addressing common misconceptions within the sex worker community [15]. NSWP recommends that these conversations be led by sex workers themselves and be framed as a form of capacity building, enabling members of the community to make informed decisions about whether or how to integrate PrEP [15, 43]. Given the history of human rights abuses against sex workers, the voluntariness of PrEP must be emphasized at all steps of the process [34]. Formative research, such as we conducted in this study, on perceived advantages and challenges of PrEP within the community can help tailor information dissemination and strategy building within each particular context. NSWP has reported concerns around difficulty in taking a pill every day and the possibility of enacted stigma or discrimination among its members, as well as additional worries about PrEP’s side effects and the belief that PrEP could decrease condom use with clients [34]. Sex worker-led organizations have also expressed concerns that PrEP may be perceived to reduce the need for comprehensive HIV prevention programs [37]. UNAIDS and other global bodies have maintained, however, that HIV prevention in any form must be situated within a comprehensive set of programs in order to effectively address sex workers’ multiple levels of vulnerability [43]. Community empowerment approaches are best situated to meet these recommendations, however the multi-level socio-structural barriers that FSW face may still be difficult to overcome without additional investments and attention to broader policy efforts to address the social, legal, and economic injustices experienced by FSW [66].

While findings from this study and prior research on barriers to PrEP among FSW indicate that a comprehensive community empowerment model would be effective in promoting uptake and adherence, there have not yet been any randomized control trials (RCTs) that attempt to establish best practices for integrating PrEP into community empowerment, indicating a clear gap in the literature [11]. Only a full RCT would allow for a complete evaluation of this model and how it may address and incorporate the barriers outlined above. Furthermore, a critical question that must be answered is how to make LAI PrEP an accessible option for FSW, particularly in lower and middle-income countries. Many of the same access challenges to oral PrEP will remain as barriers to LAI PrEP unless concerted efforts are made to overcome them, specifically how to ensure these options are not limited to higher income individuals and settings [67]. Creative and clear solutions must be developed to address the cost of LAI PrEP for FSW and other key populations globally.

A main limitation of this study was that participants were recruited through established HIV programs in each setting raising the possibility that they could have been more likely to have had previous exposure to HIV and PrEP messaging. We tried to reduce potential bias from prior exposure to HIV messaging by stratifying the sample on exposure to the intervention and we conducted this research knowing that PrEP was not widely available enough at the time for participants to have had previous exposure to PrEP messaging.

## Conclusions

As a key population at significantly high risk for HIV, the interest FSW have shown in PrEP, and LAI PrEP in particular, suggests a valuable opportunity for HIV prevention. A community empowerment approach to integrating oral and LAI PrEP as part of a menu of HIV prevention options will be critical to ensuring equitable rights-based, community-led PrEP implementation to promote optimal uptake.

## Supplementary Material

Global Inclusion

## Data Availability

Data cannot be shared publicly because of potentially sensitive information from a vulnerable population. Data are available by contacting the IRB of the Johns Hopkins Bloomberg School of Public Health for researchers who meet the criteria for access to confidential data at irb@jhsph.edu.
